# White‐tailed Deer Signpost Photoluminescence

**DOI:** 10.1002/ece3.72618

**Published:** 2025-12-14

**Authors:** Daniel R. DeRose‐Broeckert, Billy R. Hammond, Steven B. Castleberry, Gino J. D'Angelo

**Affiliations:** ^1^ Daniel B. Warnell School of Forestry and Natural Resources University of Georgia Athens Georgia USA; ^2^ Vision Sciences Laboratory, Department of Psychology University of Georgia Athens Georgia USA

**Keywords:** function, photoluminescence, signpost, ultraviolet, vision, white‐tailed deer

## Abstract

Ultraviolet (UV) induced photoluminescence is widespread in Mammalia; however, its function(s) remain unclear. Most of the research to date has focused on the surface expression of photoluminescence (e.g., pelage), described qualitatively. Here, we report a quantitative assessment of photoluminescence of white‐tailed deer (*Odocoileus virginianus,* herein deer) used for marking signposts. We analyzed 146 signposts, including 109 antler rubs on trees and 37 scent‐marking scrapes. We compared the spectra of signposts to the spectra of surrounding environmental features elicited by exposure to excitation lights peaking at 365 and 395 nm. Signposts showed significant contrast when compared to environmental backgrounds (*p* < 0.001), and the resulting photoluminescence would be visible to deer based on previously described deer visual capabilities. This research is the first quantitative description of the functional use of environmental photoluminescence by a mammal and gives new perspective about how white‐tailed deer perceive their environment and communicate.

## Graphical abstract

White‐tailed deer (*Odocoileus verginianus*) signposts exhibit photoluminescence under crepuscular conditions. The photoluminescence is visible to deer based on previously described deer visual physiology. Here we provide a quantitative description of signpost photoluminescence and discuss our findings relative to previously published criteria for the biological function of photoluminescence.

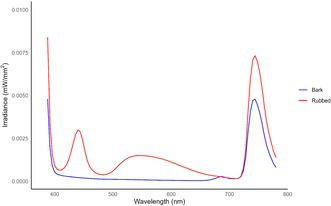

## 1 Introduction

White‐tailed deer ( *Odocoileus virginianus* , herein, deer) are a widespread, well‐studied, and intensively managed wild ungulate native to the Americas (Hewitt 2015). Many specifics are unknown, however, regarding how deer perceive their very complex environments (Newman et al. 2023). Deer rely primarily on hearing and olfaction to function (Müller‐Schwarze 1994), but vision is an essential complement to these primary senses (VerCauteren and Pipas 2003).

The study of UV‐induced photoluminescence in Mammalia has been ongoing for over a century (Reinhold et al. 2023; Travouillon et al. 2023). Photoluminescence results from photons contacting an organic object and causing a change in energy levels of lumiphores (e.g., porphyrins), resulting in the emission of light at longer wavelengths as electrons return to a ground state (Reinhold et al. 2023). To date, most research and hypotheses related to mammalian photoluminescence have focused on the presence of photoluminescence on the animal itself (anatomical photoluminescence) (Sobral and Souza‐Gudinho 2022; Toussaint et al. 2023; Travouillon et al. 2023). Little research has examined environmental photoluminescence, though theories related to anatomical photoluminescence as a form of visual camouflage within the environment have been proposed (Kohler et al. 2019).

Marshall and Johnsen (2017) proposed five criteria when assigning biological function to photoluminescence: (1) wavelengths causing photoluminescence occur naturally, (2) emitted color contrasts typical backgrounds, (3) the photoluminescent object is in a visible location, (4) the intended observer has spectral sensitivity that encompasses the range of photoluminescence, and (5) a correlation exists between photoluminescence and the animal's behavior. Several explanations have been proposed regarding the biological function of photoluminescence in Mammalia and sensitivity to UV light, including predator evasion through Batesian mimicry or camouflage (Kohler et al. 2019; Anich et al. 2021; Pynne et al. 2021), visual enhancement in low light (Kohler et al. 2019), conspecific communication (Pynne et al. 2021; Cronin and Bok 2016), and enhanced foraging, especially in snowy conditions (Cronin and Bok 2016). Although these hypotheses were often supported by data, to date, no case has been documented in which all criteria proposed by Marshall and Johnsen (2017) were met.

Deer use chemicals for olfactory communication by depositing scent at specific locations within the environment known as signposts (i.e., rubs, scrapes). Signposts facilitate both visual and olfactory communication between deer (Moore and Marchinton 1974). Scents on signposts include glandular secretions, urine, and feces (Gassett et al. 1996, 1997; Osborn et al. 2000). Male deer make rubs on vegetation by vigorously raking their antlers, removing outer bark layers (Atkeson and Marchinton 1982). Rubbing is seasonal and serves to remove antler velvet, and for adult males to advertise their presence. Rubbing is concurrent with increased sudoriferous gland activity as plasma androgen concentrations (i.e., testosterone) rise (Mirarchi et al. 1977; Kile and Marchinton 1977). Scrapes consist of a licking branch 1–2 m above ground and a depression pawed in the soil beneath the licking branch (Miller et al. 1991). Deer urinate onto their tarsal glands and onto the overturned soil (Moore and Marchinton 1974). Scent left on the licking branch is thought to originate from forehead, preorbital, and nasal gland secretions and saliva (Miller et al. 1991). Deer chew the licking branch, exposing inner wood layers. The olfactory function of signposts for conspecific communication has been investigated (Mirarchi et al. 1977; Hirth 1977; Atkeson and Marchinton 1982; Gassett et al. 1996), and the basic visual components of signposts have been described (Kile and Marchinton 1977). No study, however, has incorporated knowledge of deer vision relative to signposts.

Deer vision is adapted for heightened sensitivity in lowlight conditions (Newman et al. 2023), with short‐wavelength (SWS) cones maximally sensitive to 450–460 nm and middle‐wavelength (MWS) cones sensitive to 537 nm (Jacobs et al. 1994). The optical lens of deer lacks substantial pigments capable of filtering UV light (D'Angelo et al. 2008), and deer have been hypothesized to be visually sensitive to short wavelengths that predominate during crepuscular hours (Kieffer and Stone 2005; Cohen et al. 2014). Deer forehead glands produce various phenols and terpenes (Gassett et al. 1997), and similar compounds have been shown to photoluminesce (Aleixandre‐Tudo and du Toit 2019; Lee et al. 2013). Terpenes are produced by plants, and the cambium layer of some trees photoluminesce when exposed to UV light (Meier 2015). Lastly, deer urine contains porphyrins (Lim and Peters 1984; Neves and Galván 2020) and amino acids, both of which exhibit photoluminescence (Frohlich 2020). Therefore, we hypothesized that (1) UV light would increase the visibility of the signposts and (2) rubbed trees, urine, scraped earth, and licking branches would exhibit photoluminescence when exposed to UV light. To investigate the potential photoluminescence of deer signposts, we aimed to quantitatively measure the spectra of signposts exposed to UV wavelengths.

## 2 Methods

### 2.1 Study Site and Search Description

The study was conducted in Whitehall Forest in Athens‐Clarke County, Georgia, USA (33.89686, −83.36222). Whitehall Forest is a 337‐ha research forest located in the northeastern portion of the Georgia Piedmont physiographic region and is owned and managed by the Daniel B. Warnell School of Forestry and Natural Resources, University of Georgia.

We systematically searched for deer signposts during two time periods: September 8–October 2, 2024, and October 14–November 15, 2024. The period of September 8–October 2, 2024, represented peak rubbing activity and early scraping activity for deer in the region (Kile and Marchinton 1977). The period of October 14–November 12, 2024, represented late rubbing activity and peak scraping activity (Kile and Marchinton 1977; Alexy et al. 2001; Hearst et al. 2021). We marked deer signposts with flagging tape and a GPS point and recorded the vegetation species associated with the signpost.

### 2.2 Materials and Data Collection

Signpost spectra were collected within 1–5 days after discovery. We conducted all measurements of the spectral qualities of signposts at night and under an opaque tarp to block any incident light. Each component of a signpost (i.e., rub, licking branch, scraped earth, and urine) was exposed to a 365 nm light (Way Too Cool LLC, Glendale, Arizona, USA) and a 395 nm light (Morpilot, Milpitas, California, USA). The utilization of two wavelengths (365 and 395 nm) accounts for variable excitation of potential lumiphores, and both 365 and 395 nm are present in the atmosphere during crepuscular hours (Kieffer and Stone 2005; Théry et al. 2008). We completed two scans per component under each lighting condition (i.e., four total scans) using a PR‐650 Telescoping Spectrometer (Photo Research Inc., Chatsworth, California, USA). A scan consisted of positioning the PR‐650 35.6–45.7 cm from the target (e.g., rub, bark, scraped earth, forest floor), adjusting the focus so that the sensor was clearly centered on the target, exposing the target to UV light (395 or 365 nm), and recording irradiance values reflected/emitted by the target at each wavelength. The PR‐650 was mounted on a tripod with a custom shroud affixed to the side to reduce UV light source interaction with the sensor. Following the four scans of each signpost, we completed four scans of the untouched tree bark of the rubbed tree, or for scrapes, we scanned the surrounding substrate external to the scrape site under the same lighting conditions. We visually searched scrape sites for deer urine and scanned the urine under the same lighting conditions.

### 2.3 Analysis

We averaged the two scans under each lighting condition (i.e., 365 and 395 nm) for each signpost component (i.e., rub, licking branch, scraped earth, and urine) for direct comparison to the average of the scans for the surrounding environment component (i.e., rub versus untouched tree bark of rubbed tree) using an Excel spreadsheet (Microsoft, Redmond, WA). Because spectral data are continuous, positive, and skewed, we used a generalized linear model (GLM) with a Gamma log link (R‐core Team 2025). This model allowed for comparison of the spectra (i.e., irradiance and wavelength) of all rubs and licking branches to the spectra of all untouched bark and compared the spectra of all urine and scraped earth to all forest floor spectra. Specifically, the model allowed for treating the irradiance as a response variable to several predictor variables (i.e., rubbed/scraped = 1, unrubbed/forest floor = 0, date range (September 8–October 2 and October 14–November 12), tree species, and wavelength) to determine their individual effects on irradiance. Most of the spectra consisted of reflectance, which is visually evident by the large irradiance peak corresponding to the light source used (i.e., 365 and 395 nm). Any peaks at wavelengths longer than 400 nm were considered photoluminescence and not reflectance. To determine the statistical significance of any potential photoluminescence, we analyzed irradiance values at wavelengths between 400 and 554 nm. By limiting our analysis to 400–554 nm, we avoided reflectance peaks influencing statistical results while still remaining within deer visual sensitivity (Jacobs et al. 1994; D'Angelo et al. 2008).

## 3 Results

We scanned 109 rubs for analysis (September 8–October 2 = 57, October 14–November 12 = 52), which occurred on 20 tree species (see Table S1). We scanned 37 scrapes for analysis (September 8–October 2, 2024 = 10, October 14–November 12, 2024 = 27) with licking branches occurring on 8 tree species (see Table S2). Urine was present at 20 scrape sites, and only during the October 14–November 12 time frame. Early in the study, we determined that the PR‐650 sensor could not accurately detect licking branches because of their small size, so we did not include them in the analysis. Additionally, all scans of the overturned earth in the center of the scrape did not exhibit photoluminescence and exhibited lower average irradiance values under both lighting conditions, so we excluded overturned earth from further analysis.

**FIGURE 1:**
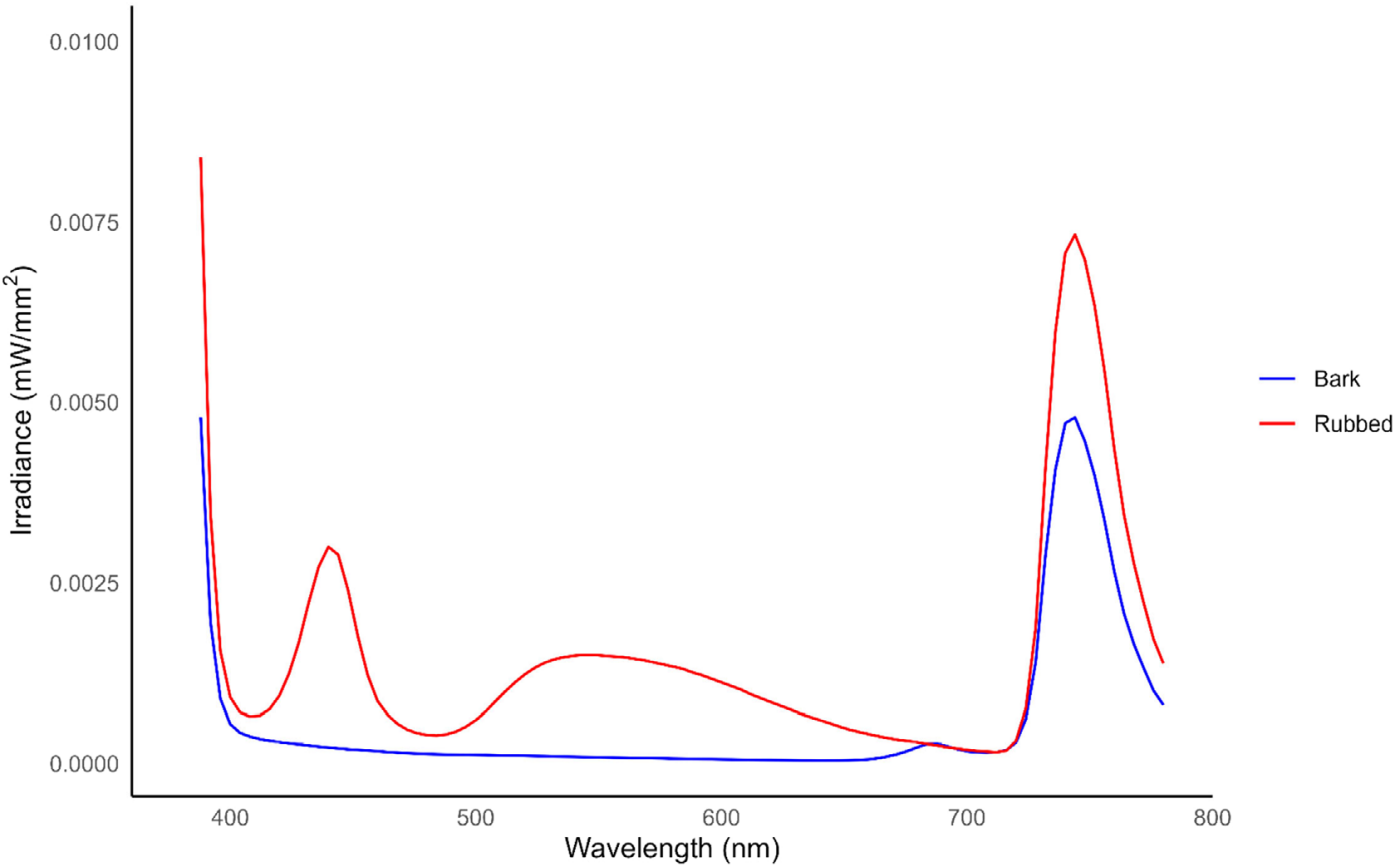
The average irradiance of rubs (*N* = 109) made by white‐tailed deer ( *Odocoileus virginianus* ) in Athens‐Clarke County, Georgia, and bark exposed to 365 nm ultraviolet (UV) light from September 8–October 2, 2024, (*N* = 57) and October 14–November 12, 2024, (*N* = 52). Rubs and tree bark were exposed to 365 nm UV light and scanned with a PR‐650 spectrophotometer. A generalized linear model was used to compare the spectra of rubbed portions of trees to their bark. Rubs had greater average irradiance values (*p* < 0.001), and exhibited photoluminescence at approximately 450 nm and 550 nm, which aligns well with white‐tailed deer short‐wave sensitive (SWS) (450–460 nm) and middle‐wave sensitive (MWS) (537 nm) cones (Jacobs et al. 1994). This indicates that rub visibility was increased in low light conditions, and that the resulting emission was visible to white‐tailed deer.

**FIGURE 2:**
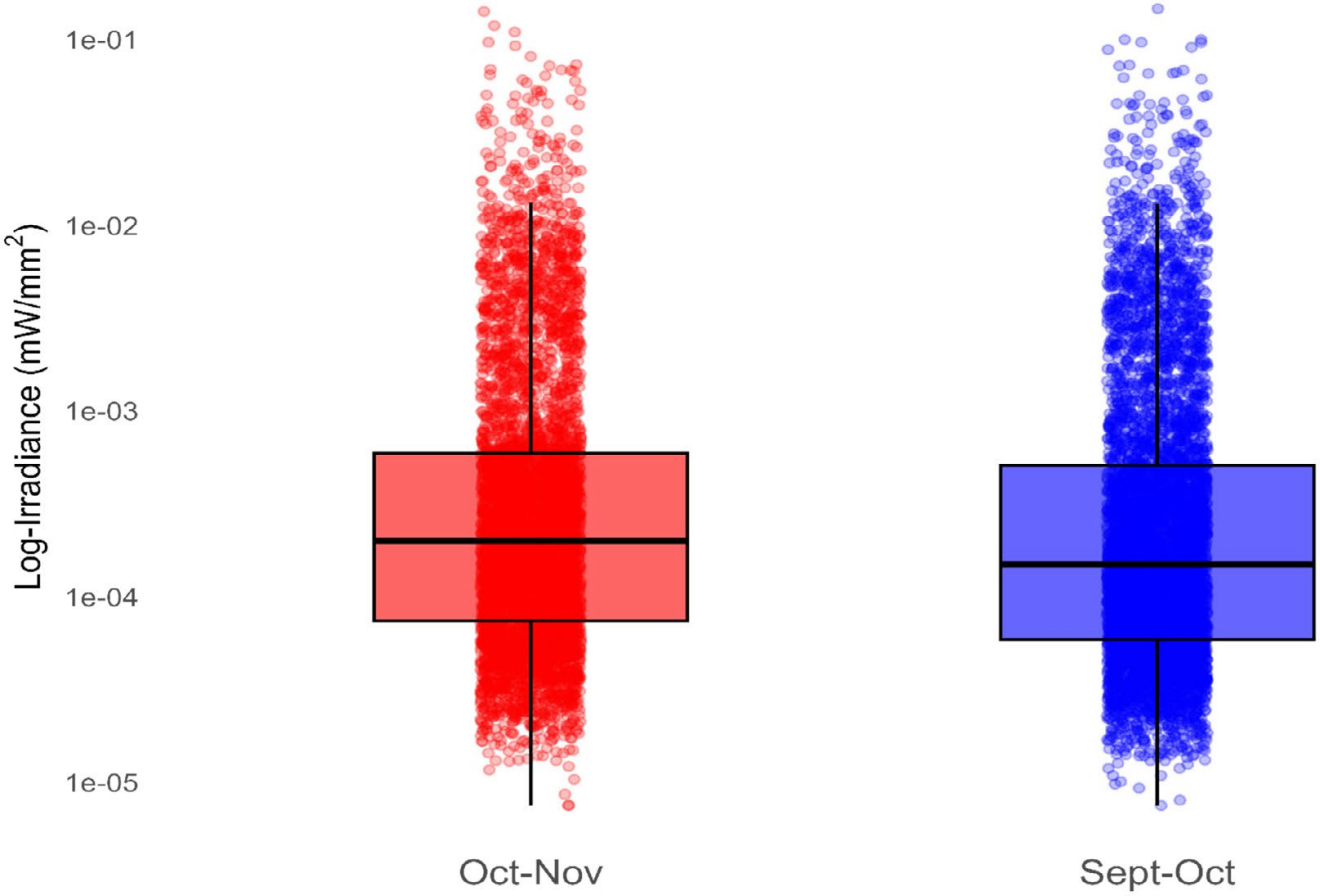
Difference in log‐irradiance of individual rub's spectra (380–780 nm) created by white‐tailed deer ( *Odocoileus virginianus* ) from September 8–October 2, 2024 (*N* = 57) and October 14–November 12, 2024, (*N* = 52) in Athens‐Clarke County, Georgia, when exposed to 365 nm ultraviolet (UV) light. Rubs were exposed to 365 nm UV light and scanned with a PR‐650 spectrophotometer to quantify rub spectral characteristics and investigate photoluminescence. Rubs created from September 8–October 2, 2024, had lower irradiance than rubs made from October 14–November 12, 2024, (*p* < 0.001). This indicates that as proximity to the breeding season increased (mid‐Nov), rub visibility increased.

Rubbed trees had greater average irradiance values than un‐rubbed trees when exposed to 365 nm (*p* < 0.001) (see Table S3) and exhibited photoluminescence peaks at 450 and 550 nm (Figure 1). Rubs created during the September–October period had lower irradiance than rubs created during the October–November timeframe (*p* < 0.001) (see Table S3) (Figure 2). Our GLM model identified a single loblolly pine ( *Pinus taeda* ) as an outlier and failed to converge, even after adjusting convergence criteria and rescaling the response variable (irradiance) (Gelman and Hill 2007). We determined that the outlier was not the result of user or equipment error and therefore wanted to retain it for analysis. In order to retain the outlier, we log‐transformed the response variable and fit our data to a linear model (LM) using ordinary least squares (OLS) (Gelman and Hill 2007). The log‐transformed LM (outlier present) yielded the same statistically significant results as the GLM (outlier absent) in that rubs had greater average irradiance values than unrubbed trees (*p* < 0.001). Since the significance of results was not altered by the presence of the outlier, we retained it in the analysis and present the results of the GLM.

**FIGURE 3:**
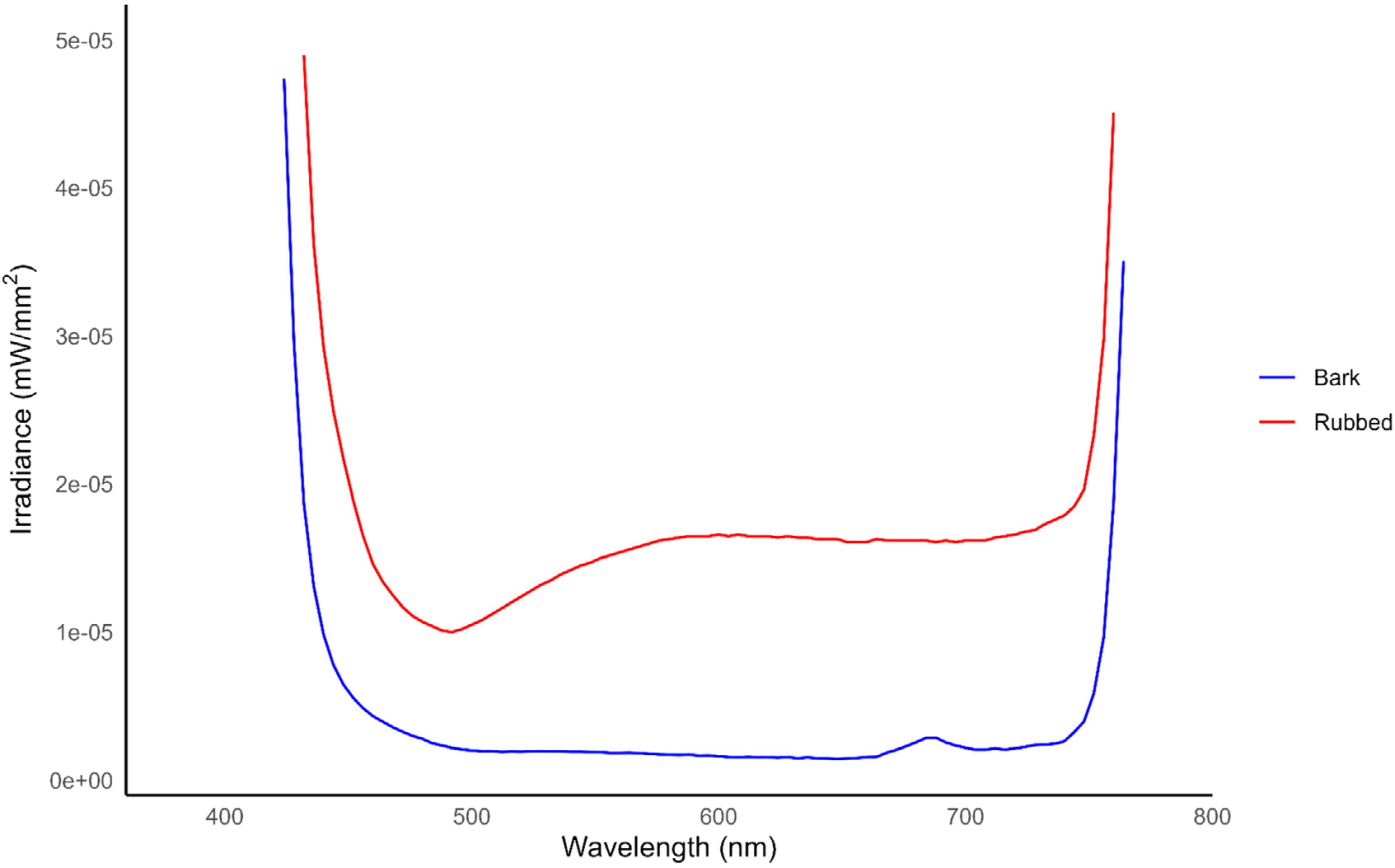
The average irradiance of rubs (*N* = 109) created by white‐tailed deer ( *Odocoileus virginianus* ) in Athens‐Clarke County, Georgia, and bark exposed to 395 nm ultraviolet (UV) light from September 8–October 2, 2024, (*N* = 57) and October 14–November 12, 2024, (*N* = 52). Rubs and tree bark were exposed to 395 nm UV light and scanned with a PR‐650 spectrophotometer. A generalized linear model was used to compare the spectra of rubbed portions of trees to their bark. Rubs had greater average irradiance values (*p* < 0.001), and exhibit photoluminescence from approximately 500 nm to 590 nm, which aligns with white‐tailed deer middle‐wave sensitive (MWS) cone (537 nm) (Jacobs et al. 1994). This indicates that rub visibility was increased in low light conditions, and that the resulting emission was visible to white‐tailed deer.

**FIGURE 4:**
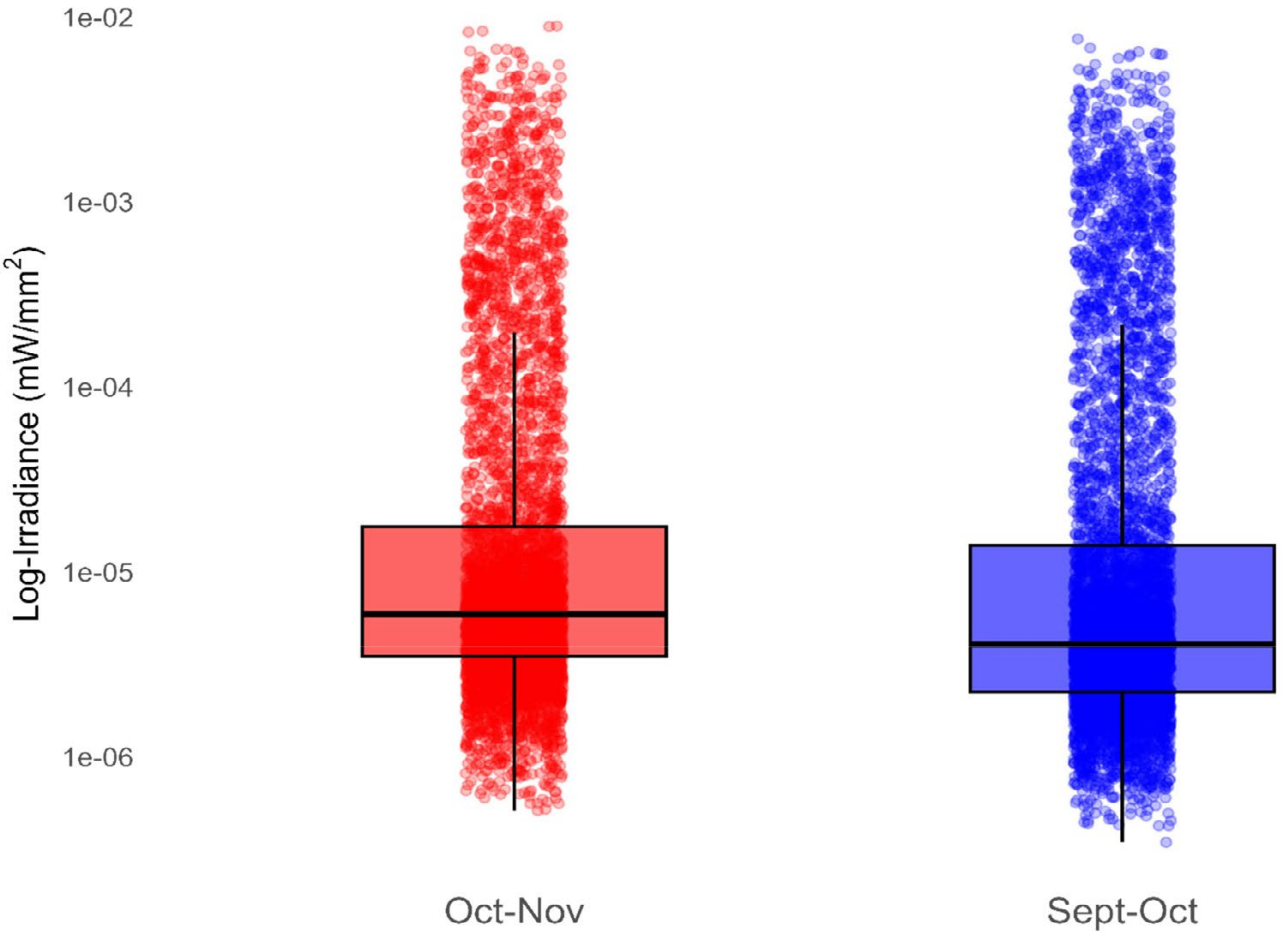
Difference in log‐irradiance of individual rub's spectra (380–780 nm) created by white‐tailed deer ( *Odocoileus virginianus* ) from September 8–October 2, 2024 (*N* = 57) and October 14–November 12, 2024, (*N* = 52) in Athens‐Clarke County, Georgia, when exposed to 395 nm ultraviolet (UV) light. Rubs were exposed to 395 nm UV light and scanned with a PR‐650 spectrophotometer to quantify rub spectral characteristics and investigate photoluminescence. Rubs created from September 8–October 2, 2024, had lower irradiance than rubs made from October 14–November 12, 2024, (*p* = 0.207). This indicates that as proximity to the breeding season increased (mid‐Nov), rub visibility increased.

Rubbed trees had greater average irradiance values than unrubbed trees when exposed to 395 nm (*p* < 0.001) (see Table S4) and exhibited photoluminescence from approximately 500–590 nm (Figure 3). Rubs created during the September–October period had lower irradiance values than rubs created during the October–November period (*p* = 0.207) (see Table S4) (Figure 4). A post hoc visual check of model assumptions revealed a single Chinese privet ( *Ligustrum sinense* ) and a single winged elm ( *Ulmus alata* ) as potential outliers (Gelman and Hill 2007). However, excluding them from our model did not alter the significance of the results, and we retained them in the analysis.

**FIGURE 5:**
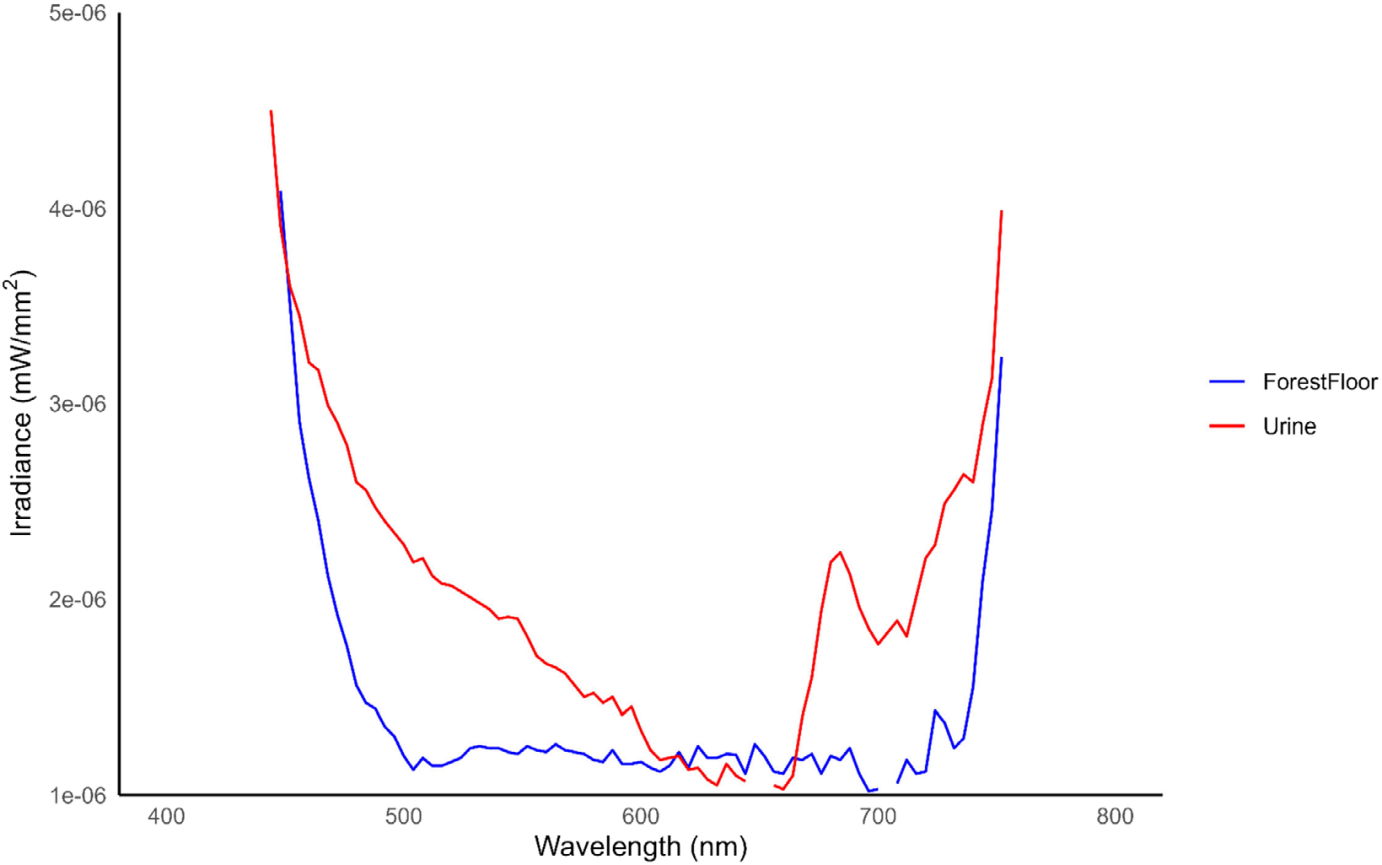
The average irradiance of white‐tailed deer ( *Odocoileus virginianus* ) urine found at scrapes (*N* = 20) and surrounding forest floor when exposed to 395 nm ultraviolet (UV) in Athens‐Clarke County, Georgia, from October 14–November 12, 2024. Urine and surrounding forest floor were exposed to 395 nm UV light and scanned with a PR‐650 spectrophotometer to quantify spectral characteristics of white‐tailed deer scrapes and investigate photoluminescence. A generalized linear model was used to compare the spectra of urine to the surrounding forest floor. Urine had greater average irradiance values than the surrounding forest floor (*p* = 0.005). Urine exhibited photoluminescence when exposed to 395 nm from approximately 480–600 nm. Although the difference in irradiance was significant, it was not visually striking since the urine spectra did not deviate far from normal distribution. However, the urine exposed to 395 nm on scrapes is likely visible to deer during low light conditions since the photoluminescence spectra does align with deer short‐wave sensitive (SWS) (450–460 nm) and middle‐wave sensitive (MWS) (537 nm) cones (Jacobs et al. 1994).

**FIGURE 6:**
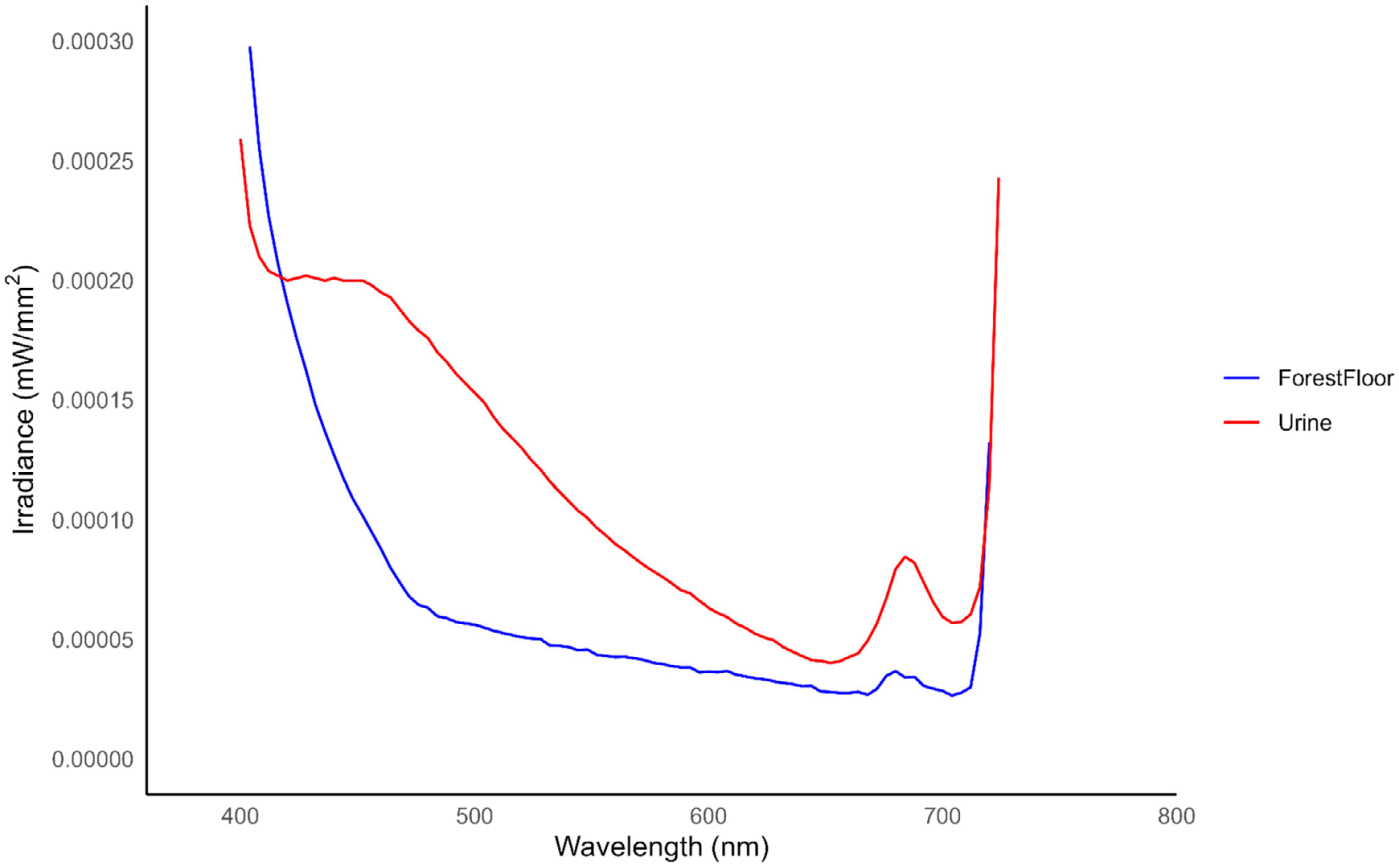
The average irradiance of white‐tailed deer ( *Odocoileus virginianus* ) urine found at scrapes (*N* = 20) and surrounding forest floor when exposed to 365 nm ultraviolet (UV) light in Athens‐Clarke County, Georgia, from October 14–November 12, 2024. Urine and the surrounding forest floor were exposed to 365 nm UV light and scanned with a PR‐650 spectrophotometer to quantify the spectral characteristics of scrapes and investigate photoluminescence. A generalized linear model was used to compare the spectra of urine to the surrounding forest floor. Urine had greater average irradiance values than the surrounding forest floor (*p* < 0.001). Urine exhibited photoluminescence from approximately 480–600 nm with a distinct peak occurring from 420 to 460 nm. The resulting emission of white‐tailed deer urine exposed to 365 nm aligns with white‐tailed deer short‐wave sensitive (SWS) (450–460 nm) cone (Jacobs et al. 1994), indicating that urine on scrapes is visible to white‐tailed deer in low‐light crepuscular conditions.

Urine associated with scrapes exposed to 395 nm had greater average irradiance values than the surrounding forest floor (*p* = 0.005) (see Table S5) and exhibited photoluminescence within approximately 480–600 nm (Figure 5). Urine exposed to 365 nm also had greater average irradiance values than the surrounding forest floor (*p* < 0.001) (see Table S6) but exhibited greater photoluminescence from 420 to 460 nm (Figure 6).

## 4 Discussion

Signpost visibility was increased by deer interactions with natural components (e.g., tree, earth via urine), and rubs created closer to the breeding season had greater irradiance values than those created earlier in the fall season. Rubs and urine found on scrapes exposed to 395 and 365 nm had greater average irradiance values (i.e., brighter) than the surrounding environment, and exhibited photoluminescence. Under both lighting conditions, the photoluminescent spectra overlapped strongly with deer short‐wave sensitive (SWS) (450–460 nm) and middle‐wave sensitive (MWS) (537 nm) cones, reaffirming that deer are visually adapted to low‐light crepuscular conditions (Jacobs et al. 1994; D'Angelo et al. 2008; Cohen et al. 2014; Newman and D'Angelo 2024). Rubs exposed to 365 nm had two distinct peaks that closely aligned with deer SWS and MWS cones, and urine exposed to 365 nm had one distinct peak aligned with deer SWS cone (Jacobs et al. 1994), indicating that signposts are uniquely enhanced for increased visibility by deer during crepuscular hours.

There are several possible causes for photoluminescence of rubs. Lignin in the cambium and inner sapwood layers of wood photoluminesce when exposed to UV light (Donaldson and Radotic 2013). Plants also produce various terpenes (Gassett et al. 1997), some of which have been shown to photoluminesce (e.g., limonene) (Lee et al. 2013). The photoluminescence of wood is so characteristic that it can be used for identification purposes (Meier 2015). Glandular secretions of the forehead gland of deer may also be responsible for the photoluminescent spectra of rubs. Gassett et al. (1997) identified 57 volatile compounds on male deer forehead glands, seven of which occurred exclusively on the forehead, including various phenols and terpenes. Both classes of compounds have been shown to exhibit photoluminescence (Lee et al. 2013; Aleixandre‐Tudo and du Toit 2019). Because white‐tailed deer do not exhibit wallowing or perfuming behaviors as seen in other cervid species (moose ( *Alces alces* ) (Miquelle 1991); elk ( *Cervus canadensis* ) (Bowyer and Kitchen 1987); fallow deer ( *Dama dama* ) (Massei and Bowyer 1999); red deer ( *Cervus elaphus* ) (Gossow and Schürholz 1974); sika deer ( *Cervus nippon* ) (Miura 1985)), we conclude that rub photoluminescence seen in this study can be attributed to forehead gland secretions (Gassett et al. 1997) or wood properties (Donaldson and Radotic 2013; Lee et al. 2013; Meier 2015; Aleixandre‐Tudo and du Toit 2019). Whether the photoluminescence is the result of deer forehead glandular secretions or wood properties, the fact remains that rubs visually contrast the surrounding environment in a way that is uniquely suited for deer vision.

The porphyrins and amino acids present in urine are the likely cause of the photoluminescent spectra we observed on scrapes (Lim and Peters 1984; Neves and Galván 2020; Frohlich 2020). Although the specific structural or chemical makeup of these compounds in deer urine is unknown, porphyrins have a range of excitation wavelengths from 385 to 398 (Reinhold et al. 2023), which aligns with part of the spectrum of the 365 nm light and the peak wavelength of the 395 nm light used in our study. Tryptophan metabolites (an essential amino acid) are present throughout the skin and pelage of many mammalian species (Reinhold et al. 2023; Travouillon et al. 2023) and urine (Oh et al. 2017). These metabolites are one of the hypothesized causes of photoluminescence on the fur and skin of several mammalian species (Pine et al. 1985; Travouillon et al. 2023; Reinhold et al. 2023). Regardless of the mode of action, the photoluminescent spectra we observed on scrapes via deer urine were within deer SWS cone visibility. More research is needed to determine the specific sources of the photoluminescence of both rubs and deer urine.

The excitation wavelengths (365 and 395 nm) used in our study are present during crepuscular hours (Kieffer and Stone 2005) when deer are most active. During crepuscular hours, ambient light is enriched with short‐UV‐blue wavelengths 345–480 nm (Théry et al. 2008). Additionally, the results of our analysis indicated a significant contrast between signposts and typical backgrounds (tree bark and forest substrate). However, structural characteristics of habitats such as canopy cover and foliage can affect what wavelengths predominate, the overall availability of visible light, and contrast levels, all of which can alter the visibility of objects to potential viewers (Goldstein 2007; Théry et al. 2008; Cuthill et al. 2019). Deer signpost activity is generally concentrated on field‐forest edges and forested trails (Kile and Marchinton 1977; Alexy et al. 2001; Huang et al. 2023), which are areas that can be characterized as being more open than closed‐canopy forests. The primary function of signposts is likely for olfactory communication (Moore and Marchinton 1974; DeYoung and Miller 2011; Ditchkoff 2011). The effectiveness of olfactory communication can be determined by levels of surface friction (i.e., vegetation structure) through the obstruction of airborne odorants (Conover 2019), indicating that olfactory signpost communication is maximized in more open locations. Based on our results, these same conditions also maximize visibility by both wavelength exposure and background contrast of signposts during UV‐blue wavelength saturated crepuscular hours.

Much of the debate pertaining to the function of photoluminescence observed in Mammalia hinges on whether the emitted spectra are visible to the target observer (Marshall and Johnsen 2017; Travouillon et al. 2023; Reinhold et al. 2023). Deer are dichromats adapted to low‐light crepuscular conditions as evidenced by the presence of SWS and MWS cones (Jacobs et al. 1994), an even distribution of rods (Staknis and Simmons 1990), and SWS cones (D'Angelo et al. 2008), and deer have been shown to be visually sensitive to shorter wavelengths (< 400 nm) (Cohen et al. 2014). The spectral signature of signposts we documented was within a range visible to deer. Rubs and urine exposed to 365 nm likely would be particularly visible to deer because the peaks of these spectra closely align with deer SWS cone types, further suggesting a possible visual relevance of signpost visibility in low‐light crepuscular conditions.

Although our methods did not directly investigate deer‐related behavioral changes associated with photoluminescence, there is evidence to suggest that a relationship exists. In Georgia, rubbing begins in late August–mid September (Kile and Marchinton 1977) as deer antlers mineralize and harden (Demarais and Strickland 2011). Early rubs are made through the process of removing the dried velvet (Moore and Marchinton 1974; Pierce et al. 2022), resulting in a combination of blood and glandular secretions remaining on the structure. Later rubs serve as both a visual and olfactory dominance display to rival males (Miller et al. 1991) as testosterone levels increase (Atkeson and Marchinton 1982; Kile and Marchinton 1977; Mirarchi et al. 1977). Our analysis indicated that rubs made earlier in the season had lower irradiance values than rubs made temporally closer to the breeding season under both lighting conditions. This suggests that rub visibility increased in tandem with increased hormone levels and known behavioral changes associated with the breeding season. Overall, the photoluminescence of rubs and urine at scrape sites could be a form of cryptic communication like that of nocturnal mammals (Olson et al. 2021; Newar et al. 2024).

It should be noted that many lumiphores are prone to photobleaching, resulting in decreased photoluminescence (Reinhold et al. 2023). Tryptophan and porphyrins in particular are known to degrade due to light exposure, with tryptophan degrading over the course of months and porphyrins within minutes (Reinhold et al. 2023; Toussaint et al. 2023). Many other lumiphores used in commercial applications have been shown to degrade at varying rates, with overall photostability depending on a wide array of environmental factors (Giroux et al. 2023). This is a likely explanation for the occurrence of outliers we observed in our study because, in a wild setting, it would be nearly impossible to know with any certainty the moment a signpost was created and therefore just as difficult to measure it within minutes of creation. A possible solution for future research targeted at controlling for temporal degradation of lumiphores present on signposts could be to observe deer in a captive setting and collect spectral measurements of signposts upon creation of the signpost.

## 5 Conclusion

Our study is the first quantification of environmental photoluminescence related to mammal ecology and the first that meets at least four of the five criteria for assigning biological function to photoluminescence proposed by Marshall and Johnsen (2017). The excitation wavelengths we used are naturally present, the emitted wavelengths contrasted with typical backgrounds, the photoluminescent portions of the signposts were in locations that maximize visibility to deer, and deer have spectral sensitivity to see the photoluminescence. Though we did not directly test for a behavioral change in deer as a result of the presence of photoluminescence, the irradiance of rubs increased at the same time as deer hormone levels increased, and behavioral changes are known to change with the progression of the breeding season. Future research investigating behavioral changes associated with signpost photoluminescence should directly manipulate photoluminescent portions of the structure so that the base color remains the same while removing the lumiphore responsible for emission (Marshall and Johnsen 2017). Future research focusing on the causal agent of the signpost photoluminescence should investigate the spectral qualities of trees and other vegetation known to be preferred by deer for signpost creation, as there could be potential for preferences related to visual attributes. Research investigating the spectral qualities of specific glandular compounds produced by deer would confirm the sources of photoluminescence documented in our study and would elucidate if signpost photoluminescence is temporally related to the breeding season. Although our results suggest a foundation for changes in deer behavior associated with signpost photoluminescence, observations of deer interacting with manipulated signposts would enhance understanding of how spectral characteristics, locations, and timing of signposts impact deer behavior.

## Author Contributions

**Daniel R. DeRose‐Broeckert:** conceptualization (equal), data curation (lead), formal analysis (lead), investigation (lead), methodology (equal), writing – original draft (lead), writing – review and editing (equal). **Billy R. Hammond:** conceptualization (supporting), formal analysis (supporting), investigation (supporting), methodology (equal), resources (lead), validation (supporting), writing – review and editing (supporting). **Steven B. Castleberry:** conceptualization (supporting), project administration (supporting), supervision (supporting), validation (supporting), writing – review and editing (supporting). **Gino J. D'Angelo:** conceptualization (equal), investigation (supporting), methodology (equal), project administration (lead), resources (supporting), supervision (supporting), writing – original draft (supporting), writing – review and editing (supporting).

## Conflicts of Interest

The authors declare no conflicts of interest.

## Supporting information


**Data S1:** ece372618‐sup‐0001‐DataS1.zip.

**Table S1:** Rubs (*N* = 109) created by white‐tailed deer ( *Odocoileus virginianus* ) from September 8 to October 2, 2024 (*N* = 57) and October 14 to November 12, 2024 (*N* = 52) in Athens‐Clarke County, Georgia. Rubs were exposed to 395 and 365 nm ultraviolet (UV) light and scanned with PR‐650 spectrophotometer to quantify spectral characteristics and investigate photoluminescence.
**Table S2:** Scrapes (*N* = 37) created by white‐tailed deer ( *Odocoileus virginianus* ) in Athens‐Clarke County, Georgia from September 8 to October 2, 2024 (*N* = 10) and October 14 to November 12, 2024 (*N* = 27) with the total for each tree species listed. All scrape sites with urine present (*N* = 20) occurred during October 14–November 12, 2024, time period. Scrapes were exposed to 395 nm and 365 nm ultraviolet (UV) light and scanned with a PR‐650 spectrophotometer to quantify spectral characteristics and investigate photoluminescence. The PR‐650 sensor was unable to detect licking branches, and scrapped earth in the middle of the scrape did not exhibit photoluminescence.
**Table S3:** Results from generalized linear model for rubs (*N* = 109) created by white‐tailed deer ( *Odocoileus virginianus* ) in Athens‐Clarke County, Georgia, when exposed to 365 nm ultraviolet (UV) light comparing irradiance of rubbed portion of trees to bark from 400 to 554 nm. Rubs were scanned with a PR‐650 spectrophotometer from September 8 to October 2, 2024, (*N* = 57) and October 14–November 12, 2024 (*N* = 52) to quantify rub spectral characteristics and investigate photoluminescence. The average rub irradiance was greater than bark (*p* < 0.001), and rubs made from September 8 to October 2, 2024, had lower average irradiance compared to rubs made from October 14 to November 12, 2024 (*p* < 0.001). Species that had a significant effect (*p* < 0.05) on irradiance relative to the intercept include American beautyberry (*Calllicarpa americana*), eastern redcedar ( *Juniperus virginiana* ), dead hardwood (spp. unkn.), hawthorn ( *Crataegus aestivalis* ), Hickory (*Carya* spp.), common persimmon ( *Diospyros virginiana* ), loblolly pine ( *Pinus taeda* ), Chinese privet ( *Ligustrum sinense* ), sweetgum ( *Liquidambar styraciflua* ), and vaccinium (*Vaccinium* spp.).
**Table S4:** Results from generalized linear model for rubs (*N* = 109) created by white‐tailed deer ( *Odocoileus virginianus* ) in Athens‐Clarke County, Georgia, when exposed to 395 nm ultraviolet (UV) light comparing irradiance of the rubbed portion of trees to bark from 400 to 554 nm. Rubs were scanned with PR‐650 spectroradiometer from September 8 to October 2, 2024 (*N* = 57) and October 14–November 12, 2024 (*N* = 52) to quantify rub spectral characteristics and investigate photoluminescence. The average rub irradiance was greater than bark (*p* < 0.001), and rubs created from September 8 to October 2, 2024, had lower average irradiance compared to rubs made from October 14 to November 12, 2024 (*p* = 0.207). Species that had a significant effect (*p* < 0.05) on irradiance relative to the intercept (American beautyberry (*Calllicarpa americana*)) include hawthorn ( *Crataegus aestivalis* ), Chinese privet ( *Ligustrum sinense* ), and winged elm ( *Ulmus alata* ).
**Table S5:** Results from generalized linear model for scrapes created by white‐tailed deer ( *Odocoileus virginianus* ) with urine present (*N* = 20) in Athens‐Clarke County, Georgia, from October 14–November 12, 2024, when exposed to 395 nm ultraviolet (UV) light comparing irradiance of deer urine to surrounding forest floor from 400 to 554 nm. Urine and adjacent forest floor were exposed to 395 nm UV light and scanned with a PR‐650 spectrophotometer to quantify scrape spectral characteristics and investigate photoluminescence. The average irradiance of white‐tailed deer urine was greater than the surrounding forest floor (*p* = 0.005).
**Table S6:** Results from generalized linear model for scrapes created by white‐tailed deer ( *Odocoileus virginianus* ) with urine present (*N* = 20) in Athens‐Clarke County, Georgia, from October 14 to November 12, 2024, when exposed to 365 nm ultraviolet (UV) light comparing irradiance of white‐tailed deer urine to the surrounding forest floor from 400 to 554 nm. Urine and adjacent forest floor were exposed to 365 nm UV light and scanned with a PR‐650 spectrophotometer to quantify scrape spectral characteristics and investigate photoluminescence. The average irradiance of white‐tailed deer urine was greater than the surrounding forest floor (*p* < 0.001).

## Data Availability

All data and information are available in Supporting Information. All rub and bark irradiance values resulting from 365 nm exposure are available in “CSV_RubData_365Light”, all rub and bark irradiance values resulting from 395 nm exposure are available in “CSV_RubData_395Light”, all urine and forest floor irradiance values resulting from 365 nm exposure are available in “CSV_UrineData_365Light”, and all urine and forest floor irradiance values resulting from 395 nm exposure are available in “CSV_UrineData_395Light”. Averages used in analysis are also available. Average rub and bark irradiance values resulting from 365 nm exposure are available in “CSV_AverageRub_365Light”, average rub and bark irradiance values resulting from 395 nm exposure are available in “CSV_AverageRub_395Light”, average urine and forest floor irradiance values resulting from 365 nm exposure are available in “CSV_AverageUrine_365Light”, and average urine and forest floor irradiance values resulting from 395 nm exposure are available in “CSV_AverageUrine_395Light”. Supplementary tables with descriptions are available in “Supplementary Tables_Signposts”.

## References

[ece372618-bib-0002] Aleixandre‐Tudo, J. L. , and W. du Toit . 2019. “The Role of UV–Visible Spectroscopy for Phenolic Compounds.” In Frontiers and New Trends in the Science of Fermented Food and Beverages, edited by R. L. Solis‐Oviedo and A. de la Cruz Pech‐Canul , 25–47. InTechOpen.

[ece372618-bib-0003] Alexy, K. J. , J. W. Gassett , D. A. Osborn , and K. V. Miller . 2001. “Remote Monitoring of Scraping Behaviors of a Wild Population of White‐Tailed Deer.” Wildlife Society Bulletin 29: 873–878. https://www.jstor.org/stable/3784414.

[ece372618-bib-0004] Anich, P. S. , S. Anthony , M. Carlson , et al. 2021. “Biofluorescence in the Platypus (*Orinthorhynchus Anatinus*).” Mammalia 85, no. 2: 179–181. 10.1515/mammalia-2020-0027.

[ece372618-bib-0005] Atkeson, T. D. , and R. L. Marchinton . 1982. “Forehead Glands in White‐Tailed Deer.” Journal of Mammalogy 63, no. 4: 613–617. https://www.jstor.org/stable/1380266.

[ece372618-bib-0006] Bowyer, T. R. , and D. W. Kitchen . 1987. “Significance of Scent‐Marking by Roosevelt Elk.” Journal of Mammalogy 86, no. 2: 418–423. 10.2307/1381489.

[ece372618-bib-0007] Cohen, B. S. , D. A. Osborn , G. R. Gallagher , R. J. Warren , and K. V. Miller . 2014. “Behavioral Measure of the Light‐Adapted Visual Sensitivity of White‐Tailed Deer.” Wildlife Society Bulletin 38, no. 3: 480–485. 10.1002/wsb.438.

[ece372618-bib-0008] Conover, M. R. 2019. Predator–Prey Dynamics: The Role of Olfaction, 82–124. CRC Press.

[ece372618-bib-0009] Cronin, T. W. , and M. J. Bok . 2016. “Photoreception and Vision in the Ultraviolet.” Journal of Experimental Biology 219: 2790–2801. 10.1242/jeb.128769. 27655820

[ece372618-bib-0010] Cuthill, I. C. , S. R. Matchette , and N. E. Scott‐Samual . 2019. “Camouflage in a Dynamic World.” Current Opinion in Behavioral Sciences 30: 109–115. 10.1016/j.cobeha.2019.07.007.

[ece372618-bib-0011] D'Angelo, G. J. , A. Glasser , M. Wendt , et al. 2008. “Visual Specialization of an Herbivore Prey Species, the White‐Tailed Deer.” Canadian Journal of Zoology 86: 735–743. 10.1139/Z08-050.

[ece372618-bib-0012] Demarais, S. , and B. K. Strickland . 2011. “Antlers.” In Biology and Management of White‐Tailed Deer, edited by D. G. Hewitt , 107–146. CRC Press.

[ece372618-bib-0013] DeYoung, R. W. , and K. V. Miller . 2011. “White‐Tailed Deer Behavior.” In Biology and Management of White‐Tailed Deer, edited by D. G. Hewitt , 311–351. CRC Press.

[ece372618-bib-0014] Ditchkoff, S. S. 2011. “Anatomy and Physiology.” In Biology and Management of White‐Tailed Deer, edited by D. G. Hewitt , 43–73. CRC Press.

[ece372618-bib-0015] Donaldson, L. A. , and K. Radotic . 2013. “Fluorescence Lifetime Imaging of Lignin Autofluorescence in Normal and Compression Wood.” Journal of Microscopy 251, no. 2: 178–187. 10.1111/jmi.12059. 23763341

[ece372618-bib-0017] Frohlich, J. 2020. “Rats and Mice.” In Ferrets, Rabbits, and Rodents: Clinical Medicine and Surgery, edited by K. Quesenberry , C. Mans , and C. Orcutt , 345–367. Imprint: Saunders.

[ece372618-bib-0018] Gassett, J. W. , D. P. Wiesler , A. G. Baker , et al. 1996. “Volatile Compounds From Interdigital Gland of Male White‐Tailed Deer (*Odocoileus virginianus*).” Journal of Chemical Ecology 22, no. 9: 1689–1696. 24226480 10.1007/BF02272407

[ece372618-bib-0019] Gassett, J. W. , D. P. Wiesler , A. G. Baker , et al. 1997. “Volatile Compounds From the Forehead Region of Male White‐Tailed Deer ( *Odocoileus virginianus* ).” Journal of Chemical Ecology 23, no. 3: 569–578. 10.1007/BF02272407 24226480

[ece372618-bib-0020] Gelman, A. , and J. Hill . 2007. “Generalize Linear Models.” In Data Analysis Using Regression and Multilevel/Hierarchical Models, 109–132. Cambridge University Press.

[ece372618-bib-0021] Giroux, M. , Z. Zahra , O. A. Salawu , R. M. Burgess , K. T. Ho , and A. S. Adeleye . 2023. “Assessing the Environmental Effects Related to Quantum Dot Structure, Function, Synthesis and Exposure.” Environmental Science Nano 9, no. 3: 867–910. 10.1039/d1en00712b. PMC8992011 35401985

[ece372618-bib-0022] Goldstein, B. E. 2007. Sensation and Perception, Seventh Edition, 151–190. Thomson Learning, Inc.

[ece372618-bib-0023] Gossow, H. , and G. Schürholz . 1974. “Social Aspects of Wallowing Behaviour in Red Deer Herds.” Zeitschrift für Tierpsychologie 34: 329–336.

[ece372618-bib-0024] Hearst, S. , S. Streeter , H. Sharron , et al. 2021. “Scraping Network Analysis: A Method to Explore Complex White‐Tailed Deer Mating Systems.” Southeastern Naturalist 20, no. 1: 192–211. 10.1656/058.020.0122.

[ece372618-bib-0025] Hewitt, D. G. 2015. “Hunters and the Conservation and Management of White‐Tailed Deer ( *Odocoileus virginianus* ).” International Journal of Environmental Studies 72, no. 5: 839–849. 10.1080/00207233.2015.1073473.

[ece372618-bib-0026] Hirth, D. H. 1977. “Social Behavior of White‐Tailed Deer in Relation to Habitat.” Wildlife Monographs 53: 3–55. https://www.jstor.org/stable/3830446.

[ece372618-bib-0027] Huang, M. H. J. , S. Demarais , A. Banda , et al. 2023. “Expanding CWD Disease Surveillance Options Using Environmental Contamination at Deer Signposts.” Ecological Solutions and Evidence 5: e12298. 10.1002/2688-8319.12298.

[ece372618-bib-0028] Jacobs, G. H. , J. F. Deegan II , J. Neitz , B. P. Murphy , K. V. Miller , and R. L. Marchinton . 1994. “Electrophysiological Measurements of Spectral Mechanisms in the Retinas of Two Cervids: White‐Tailed Deer (*Odocoileus Virginianus*) and Fallow Deer (*Dama Dama*).” Journal of Comparative Physiology 174: 551–557. 10.1007/BF00217375. 8006855

[ece372618-bib-0029] Kieffer, H. H. , and T. C. Stone . 2005. “The Spectral Irradiance of the Moon.” Astronomical Journal 129: 2887–2901.

[ece372618-bib-0030] Kile, T. L. , and R. L. Marchinton . 1977. “White‐Tailed Deer Rubs and Scrapes: Spatial, Temporal and Physical Characteristics and Social Role.” American Midland Naturalist 97, no. 2: 257–266. https://www.jstor.org/stable/2425092.

[ece372618-bib-0031] Kohler, A. M. , E. R. Olson , J. G. Martin , and P. S. Anich . 2019. “Ultraviolet Fluorescence Discovered in New World Flying Squirrels (*Glaucomys*).” Journal of Mammalogy 100, no. 1: 21–30. 10.1093/jmammal/gyy177.

[ece372618-bib-0033] Lee, H. J. , A. Laskin , J. Laskin , and S. A. Nizkorodov . 2013. “Excitation‐Emission Spectra and Fluorescence Quantum Yields for Fresh and Aged Biogenic Secondary Organic Aerosols.” Environmental Science & Technology 47: 5763–5770. 10.1021/es400644c. 23663151

[ece372618-bib-0034] Lim, C. K. , and T. J. Peters . 1984. “Urine and Fecal Porphyrin Profiles by Reversed‐Phase High‐Performance Liquid Chromatography in the Porphyrins.” Clinica Chimica Acta 139: 55–63. 10.1016/0009-8981(84)90192-x 6723073

[ece372618-bib-0035] Marshall, J. , and S. Johnsen . 2017. “Fluorescence as a Means of Colour Signal Enhancement.” Philosophical Transactions of the Royal Society, B: Biological Sciences 372: 20160335. 10.1098/rstb.2016.0335. PMC5444056 28533452

[ece372618-bib-0036] Massei, G. , and R. T. Bowyer . 1999. “Scent Marking in Fallow Deer: Effects of Lekking Behavior on Rubbing and Wallowing.” Journal of Mammalogy 80, no. 2: 633–638.

[ece372618-bib-0037] Meier, E. 2015. “Fluorescence: A Secret Weapon in Wood Identification.” The Wood Database. Accessed 6 April 2025. www.wood‐database.com/wood‐articles/fluorescence‐a‐secret‐weapon‐in‐wood‐identification/.

[ece372618-bib-0038] Miller, K. V. , R. L. Marchinton , and P. B. Bush . 1991. “Signpost Communication by White‐Tailed Deer: Research Since Calgary.” Applied Animal Behaviour Science 29: 195–204.

[ece372618-bib-0039] Miquelle, D. G. 1991. “Are Moose Mice? The Function of Scent Urination in Moose.” American Naturalist 138, no. 2: 460–477. 10.1086/285226.

[ece372618-bib-0040] Mirarchi, R. E. , P. F. Scanlon , R. L. Kirkpatrick , and C. B. Schreck . 1977. “Androgen Levels and Antler Development in Captive and Wild White‐Tailed Deer.” Journal of Wildlife Management 41, no. 2: 178–183. https://www.jstor.org/stable/3800591.

[ece372618-bib-0041] Miura, S. 1985. “Why Do Male Sika Deer Wallow During the Rut.” Journal of Ethology 3: 73–75.

[ece372618-bib-0042] Moore, W. G. , and R. L. Marchinton . 1974. “Marking Behavior and Its Social Function in White‐Tailed Deer.” In The Behaviour of Ungulates and Its Relation to Management. The Papers of an International Symposium Held, edited by V. Geist and F. Walther , 447–456. University of Calgary, Alberta.

[ece372618-bib-0043] Müller‐Schwarze, D. 1994. “The Senses of Deer.” In The Wildlife Series: Deer, edited by D. Gerlach , S. Atwater , and J. Schnell , 58–65. Stackpole Books.

[ece372618-bib-0044] Neves, A. C. O. , and I. Galván . 2020. “Models for Human Porphyrias: Have Animals in the Wild Been Overlooked?” Bio Essays 42: 2000155. 10.1002/bies.202000155. 33155299

[ece372618-bib-0045] Newar, S. L. , I. Schneiderová , B. Hughes , and J. Bowman . 2024. “Ultrasound and Ultraviolet: Crypsis Gliding Mammals.” PeerJ 12: e:17048. 10.7717/peerj.17048. 38549780 PMC10977092

[ece372618-bib-0046] Newman, B. A. , and G. J. D'Angelo . 2024. “A Review of Cervidae Visual Ecology.” Animals 14, no. 3: 420. 10.3390/ani14030420. 38338063 PMC10854973

[ece372618-bib-0047] Newman, B. A. , J. R. Dyal , K. V. Miller , M. J. Cherry , and G. J. D'Angelo . 2023. “Influence of Visual Perception on Movement Decisions by an Ungulate Prey Species.” Biology Open 12: bio059932. 10.1242/bio.059932. 37843403 PMC10602006

[ece372618-bib-0049] Oh, J. S. , H. S. Seo , K. H. Kim , H. Pyo , B. C. Chung , and J. Lee . 2017. “Urinary Profiling of Tryptophan and Its Related Metabolites in Patients With Metabolic Syndrome by Liquid Chromatography‐Electrospray Ionization/Mass Spectrometry.” Analytical and Bioanalytical Chemistry 409: 5501–5512. 28710517 10.1007/s00216-017-0486-4

[ece372618-bib-0050] Olson, E. R. , M. R. Carlson , V. M. Sadagopa Ramanujam , et al. 2021. “Vivid Biofluorescence Discovered in the Nocturnal Springhare (Pedetidae).” Scientific Reports 11: 4125. 10.1038/s41598-021-83588-0. 33603032 PMC7892538

[ece372618-bib-0051] Osborn, D. A. , K. V. Miller , D. M. Hoffman , W. H. Dickerson , J. W. Gassett , and C. F. Quist . 2000. “Morphology of the White‐Tailed Deer Tarsal Gland.” Acta Theriologica 45: 117–122.

[ece372618-bib-0052] Pierce, R. A., II , J. Sumners , and E. Flinn . 2022. Antler Development in White‐Tailed Deer: Implications for Management. University of Missouri Extension G9486.

[ece372618-bib-0053] Pine, R. H. , J. E. Rice , J. E. Bucher , D. H. Tank Jr. , and A. M. Greenhall . 1985. “Labile Pigments and Fluorescent Pelage in Didelphid Marsupials.” Mammalia 49: 249–256.

[ece372618-bib-0054] Pynne, J. T. , S. B. Castleberry , L. M. Conner , et al. 2021. “Ultraviolet Biofluorescence in Pocket Gophers.” American Midland Naturalist 186, no. 1: 150–155. 10.1674/0003-0031-186.1.150.

[ece372618-bib-0055] R‐core Team . 2025. R: A Language and Environment for Statistical Computing, Version 4.4.0. R Foundation for Statistical Computing. https://www.r‐project.org/.

[ece372618-bib-0057] Reinhold, L. M. , T. L. Rymer , K. M. Helgen , and D. T. Wilson . 2023. “Photoluminescence in Mammal Fur: 111 Years of Research.” Journal of Mammalogy 104, no. 4: 892–906. 10.1093/jmammal/gyad027. 37545668 PMC10399922

[ece372618-bib-0058] Sobral, G. , and F. Souza‐Gudinho . 2022. “Fluorescence and UV–Visible Reflectance in the Fur of Several Rodentia Genera.” Scientific Reports 12: 12293. 10.1038/s41598-022-15952-7. 35853976 PMC9296623

[ece372618-bib-0059] Staknis, M. A. , and D. M. Simmons . 1990. “Ultrastructural Evaluation of the Eastern White‐Tailed Deer Retina for Color Perception.” Journal of the Pennsylvania Academy of Science 64, no. 1: 8–10.

[ece372618-bib-0060] Théry, M. , S. Pincebourde , and F. Feer . 2008. “Dusk Light Environment Optimize Visual Perception of Conspecifics in a Crepuscular Horned Beetle.” Behavioral Ecology 19: 627–632. 10.1093/beheco/arn024.

[ece372618-bib-0061] Toussaint, S. L. D. , J. Ponstein , M. Thoury , et al. 2023. “Fur Glowing Under Ultraviolet: In Situ Analysis of Porphyrin Accumulation in the Skin Appendages of Mammals.” Integrative Zoology 18: 15–26. 10.1111/1749-4877.12655. 35500584

[ece372618-bib-0062] Travouillon, K. J. , C. Cooper , J. T. Bouzin , L. S. Umbrello , and S. W. Lewis . 2023. “All‐a‐Glow: Spectral Characteristics Confirm Widespread Fluorescence for Mammals.” Royal Society Open Science 10: 230325. 10.1098/rsos.230325. 37800154 PMC10548093

[ece372618-bib-0063] VerCauteren, K. C. , and M. J. Pipas . 2003. “A Review of Color Vision in White‐Tailed Deer.” Wildlife Society Bulletin 31, no. 3: 684–691. http://www.jstor.com/stable/3784587.

