# Supplementary material for: White‐tailed Deer Signpost Photoluminescence
Source: Ecol Evol. 2025 Dec 14;15(12):e72618. doi: 10.1002/ece3.72618 (PMC12703122; doi:10.1002/ece3.72618)
Supplement: Supplementary file 2 — Table S1: Rubs (N = 109) created by white‐tailed deer ( Odocoileus virginianus ) from September 8 to October 2, 2024 (N = 57) and October 14 to November 12, 2024 (N = 52) in Athens‐Clarke County, Georgia. Rubs were exposed to 395 and 365 nm ultraviolet (UV) light and scanned with PR‐650 spectrophotometer to quantify spectral characteristics and investigate photoluminescence. Table S2: Scrapes (N = 37) created by white‐tailed deer ( Odocoileus virginianus ) in Athens‐Clarke County, Georgia from September 8 to October 2, 2024 (N = 10) and October 14 to November 12, 2024 (N = 27) with the total for each tree species listed. All scrape sites with urine present (N = 20) occurred during October 14–November 12, 2024, time period. Scrapes were exposed to 395 nm and 365 nm ultraviolet (UV) light and scanned with a PR‐650 spectrophotometer to quantify spectral characteristics and investigate photoluminescence. The PR‐650 sensor was unable to detect licking branches, and scrapped earth in the middle of the scrape did not exhibit photoluminescence. Table S3: Results from generalized linear model for rubs (N = 109) created by white‐tailed deer ( Odocoileus virginianus ) in Athens‐Clarke County, Georgia, when exposed to 365 nm ultraviolet (UV) light comparing irradiance of rubbed portion of trees to bark from 400 to 554 nm. Rubs were scanned with a PR‐650 spectrophotometer from September 8 to October 2, 2024, (N = 57) and October 14–November 12, 2024 (N = 52) to quantify rub spectral characteristics and investigate photoluminescence. The average rub irradiance was greater than bark (p < 0.001), and rubs made from September 8 to October 2, 2024, had lower average irradiance compared to rubs made from October 14 to November 12, 2024 (p < 0.001). Species that had a significant effect (p < 0.05) on irradiance relative to the intercept include American beautyberry (Calllicarpa americana), eastern redcedar ( Juniperus virginiana ), dead hardwood (spp. unkn.), hawthorn ( Crataegus aesti [file ECE3-15-e72618-s002.docx]

| **Supplementary Table 1.** Rubs (N = 109) created by white-tailed deer (*Odocoileus virginianus*) from September 8-October 2, 2024 (N = 57) and October 14-November 12, 2024 (N=52) in Athens-Clarke County, Georgia. Rubs were exposed to 395 nm and 365 nm ultraviolet (UV) light and scanned with PR-650 spectrophotometer to quantify spectral characteristics and investigate photoluminescence. | | |
| --- | --- | --- |
| **Species** | **N** | **Time period** |
| American beech (*Fagus grandifolia*) | 5 | Sept-Oct |
| Eastern redcedar (*Juniperus virginiana*) | 3 |  |
| Dead Hardwood (spp. unkn.) | 1 |  |
| Flowering dogwood (*Cornus floridana*) | 3 |  |
| Elaeagnus (*Elaeagnus* spp.) | 11 |  |
| Hickory (*Carya* spp.) | 1 |  |
| Ostrya (*Ostrya virginiana*) | 6 |  |
| Loblolly pine (*Pinus taeda*) | 5 |  |
| Red maple (*Acer* *rubrum*) | 1 |  |
| Silver maple (*Acer saccharinum*) | 1 |  |
| Rusty blackhaw (*Viburnum rufidulum*) | 1 |  |
| Sweetgum (*Liquidambar styraciflua*) | 1 |  |
| Vaccinium (*Vaccinium* spp.) | 1 |  |
| Winged elm (*Ulmus alata*) | 17 |  |
| American beautyberry (*Callicarpa americana*) | 1 | Oct-Nov |
| Boxelder (*Acer negundo*) | 1 |  |
| Carolina silverbell (*Halesia carolina*) | 4 |  |
| Eastern redcedar (*Juniperus virginiana*) | 3 |  |
| Flowering dogwood (*Cornus florida*) | 1 |  |
| Elaeagnus (*Elaeagnus* spp.) | 3 |  |
| Hawthorn (*Crataegus aestivalis*) | 1 |  |
| Ostrya (*Ostrya virginiana*) | 9 |  |
| Common persimmon (*Diospyros virginiana*) | 2 |  |
| Loblolly pine (*Pinus taeda*) | 22 |  |
| Chinese privet (*Ligustrum sinense*) | 4 |  |
| Winged elm (*Ulmus alata*) | 1 |  |
|  |  |  |

| **Supplementary Table 2.** Scrapes (N = 37) created by white-tailed deer (*Odocoileus virginianus*) in Athens-Clarke County, Georgia from September 8-October 2, 2024 (N = 10) and October 14-November 12, 2024 (N = 27) with the total for each tree species listed. All scrape sites with urine present (N = 20) occurred during October 14-November 12, 2024, time period. Scrapes were exposed to 395 nm and 365 nm ultraviolet (UV) light and scanned with a PR-650 spectrophotometer to quantify spectral characteristics and investigate photoluminescence. The PR-650 sensor was unable to detect licking branches, and scrapped earth in the middle of the scrape did not exhibit photoluminescence. | | |
| --- | --- | --- |
| **Species** | **N** | **Time period** |
| American beech (*Fagus grandifolia*) | 3 | Sept-Oct |
| Eastern redcedar (*Juniperus virginiana*) | 1 |  |
| Ostrya (*Ostrya virginiana*) | 2 |  |
| Sweetgum (*Liquidambar styraciflua*) | 1 |  |
| Vaccinium (*Vaccinium* spp.) | 1 |  |
| Winged elm (*Ulmus alata*) | 2 |  |
| American beech (*Fagus grandifolia*) | 9 | Oct-Nov |
| Elaeagnus (*Elaeagnus* spp.) | 5 |  |
| Hickory (*Carya* spp.) | 1 |  |
| Ostrya (*Ostrya virginiana*) | 5 |  |
| Chinese privet (*Ligustrum sinense*) | 5 |  |
| Sweetgum (*Liquidambar styraciflua*) | 1 |  |
| Winged elm (*Ulmus alata*) | 1 |  |

| **Supplementary Table 3.** Results from generalized linear model for rubs (N = 109) created by white-tailed deer (*Odocoileus virginianus*) in Athens-Clarke County, Georgia, when exposed to 365 nm ultraviolet (UV) light comparing irradiance of rubbed portion of trees to bark from 400-554 nm. Rubs were scanned with a PR-650 spectrophotometer from September 8-October 2, 2024, (N = 57) and October 14-November 12, 2024 (N = 52) to quantify rub spectral characteristics and investigate photoluminescence. The average rub irradiance was greater than bark (p < 0.001), and rubs made from September 8-October 2, 2024, had lower average irradiance compared to rubs made from October 14-November 12, 2024 (p < 0.001). Species that had a significant effect (p<0.05) on irradiance relative to the intercept include (American beautyberry (*Calllicarpa americana*), eastern redcedar (*Juniperus virginiana*), dead hardwood (spp. unkn.), hawthorn (*Crataegus aestivalis*), Hickory (*Carya* spp.), common persimmon (*Diospyros virginiana*), loblolly pine (*Pinus taeda*), Chinese privet (*Ligustrum sinense*), sweetgum (*Liquidambar styraciflua*), and vaccinium (*Vaccinium* spp.). | | | | |
| --- | --- | --- | --- | --- |
| **Coefficient** | **Estimate** | **Std. Error** | **t** | **p** |
| Rubbed | 0.948 | 0.036 | 26.341 | <0.001 |
| September-October | -0.164 | 0.048 | 3.368 | <0.001 |
| Intercept (Am. Beautyberry (*Callicarpa americana*)) | 6.306 | 0.226 | 27.839 | <0.001 |
| American beech (*Fagus grandifolia*) | -0.021 | 0.182 | -0.116 | 0.907 |
| Boxelder (*Acer negundo*) | 0.131 | 0.210 | 0.627 | 0.530 |
| Carolina silverbell (*Halesia carolina*) | -0.075 | 0.166 | -0.455 | 0.649 |
| Eastern redcedar (*Junipurus virginiana*) | 0.485 | 0.165 | 2.938 | 0.003 |
| Dead Hardwood (spp. unkn.) | 0.838 | 0.261 | 3.203 | 0.001 |
| Flowering dogwood (*Cornus floridana*) | 0.208 | 0.172 | 1.212 | 0.225 |
| Elaeagnus (*Elaeagnus* spp.) | 0.171 | 0.158 | 1.082 | 0.279 |
| Hawthorn (*Crataegus aestivalis*) | -1.323 | 0.258 | .5.128 | <0.001 |
| Hickory (*Carya* spp.) | 0.556 | 0.261 | 2.126 | 0.033 |
| Ostrya (*Ostrya virginiana*) | 0.120 | 0.155 | 0.774 | 0.439 |
| Common persimmon (*Diospyros virginiana*) | 0.367 | 0.182 | 2.020 | 0.043 |
| Loblolly pine (*Pinus taeda*) | 0.316 | 0.151 | 2.085 | 0.037 |
| Chinese privet (*Ligustrum sinense*) | 0.119 | 0.171 | 0.694 | 0.487 |
| Red maple (*Acer* *rubrum*) | -0.362 | 0.261 | -1.383 | 0.166 |
| Rusty blackhaw (*Viburnum rufidulum*) | -0.382 | 0.215 | -1.774 | 0.076 |
| Silver maple (*Acer saccharinum*) | -0.163 | 0.261 | -0.625 | 0.532 |
| Sweetgum (*Liquidambar styraciflua*) | 0.623 | 0.261 | 2.382 | 0.017 |
| Vaccinium (*Vaccinium* spp.) | -0.640 | 0.261 | -2.444 | 0.014 |
| Winged elm (*Ulmus alata*) | 0.090 | 0.162 | 0.560 | 0.572 |
|  |  |  |  |  |

| **Supplementary Table 4.** Results from generalized linear model for rubs (N = 109) created by white-tailed deer (*Odocoileus virginianus*) in Athens-Clarke County, Georgia, when exposed to 395 nm ultraviolet (UV) light comparing irradiance of the rubbed portion of trees to bark from 400-554 nm. Rubs were scanned with PR-650 spectroradiometer from September 8-October 2, 2024 (N = 57) and October 14-November 12, 2024 (N = 52) to quantify rub spectral characteristics and investigate photoluminescence. The average rub irradiance was greater than bark (p<0.001), and rubs created from September 8-October 2, 2024, had lower average irradiance compared to rubs made from October 14-November 12, 2024 (p=0.207). Species that had a significant effect (p<0.05) on irradiance relative to the intercept (American beautyberry (*Calllicarpa americana*)) include hawthorn (*Crataegus aestivalis*), Chinese privet (*Ligustrum sinense*), and winged elm (*Ulmus alata*). | | | | |
| --- | --- | --- | --- | --- |
| **Coefficient** | **Estimate** | **Std. Error** | **t** | **p** |
| Rubbed | 1.023 | 0.080 | 12.704 | <0.001 |
| September-October | -0.138 | 0.109 | -1.261 | 0.207 |
| Intercept (Am. Beautyberry (*Callicarpa americana*) | 6.050 | 0.508 | 11.907 | <0.001 |
| American beech (*Fagus grandifolia*) | -0.087 | 0.410 | -0.213 | 0.831 |
| Boxelder (*Acer negundo*) | 0.072 | 0.473 | 0.153 | 0.878 |
| Carolina silverbell (*Halesia carolina*) | -0.097 | 0.374 | -0.260 | 0.795 |
| Eastern redcedar (*Juniperus virginiana*) | 0.485 | 0.372 | 1.305 | 0.191 |
| Dead Hardwood (spp. unkn.) | 0.697 | 0.590 | 1.181 | 0.237 |
| Flowering dogwood (*Cornus florida*) | 0.158 | 0.387 | 0.408 | 0.683 |
| Elaeagnus (*Elaeagnus* spp.) | 0.125 | 0.357 | 0.351 | 0.725 |
| Hawthorn (*Crataegus aestivalis*) | -1.402 | 0.581 | -2.411 | 0.015 |
| Hickory (*Carya* spp.) | 0.513 | 0.590 | 0.869 | 0.384 |
| Ostrya (*Ostrya virginiana*) | 0.107 | 0.349 | 0.306 | 0.759 |
| Common persimmon (*Diospyros virginiana*) | 0.399 | 0.410 | 0.973 | 0.330 |
| Loblolly pine (*Pinus taeda*) | 0.284 | 0.341 | 0.832 | 0.405 |
| Chinese privet (*Ligustrum sinense*) | 1.663 | 0.374 | 4.439 | <0.001 |
| Red maple (*Acer* *rubrum*) | -0.468 | 0.590 | -0.794 | 0.427 |
| Rusty blackhaw (*Viburnum rufidulum*) | -0.463 | 0.486 | -0.954 | 0.340 |
| Silver maple (*Acer saccharinum*) | -0.287 | 0.590 | -0.487 | 0.626 |
| Sweetgum (*Liquidambar styraciflua*) | 0.587 | 0.590 | 0.994 | 0.320 |
| Vaccinium (*Vaccinium* spp.) | -0.699 | 0.590 | -1.185 | 0.235 |
| Winged elm (*Ulmus alata*) | 1.393 | 0.364 | 3.822 | <0.001 |
|  |  |  |  |  |

| **Supplementary Table 5.**  Results from generalized linear model for scrapes created by white-tailed deer (*Odocoileus virginianus*) with urine present (N = 20) in Athens-Clarke County, Georgia, from October 14-November 12, 2024, when exposed to 395 nm ultraviolet (UV) light comparing irradiance of deer urine to surrounding forest floor from 400-554 nm. Urine and adjacent forest floor were exposed to 395 nm UV light and scanned with a PR-650 spectrophotometer to quantify scrape spectral characteristics and investigate photoluminescence. The average irradiance of white-tailed deer urine was greater than the surrounding forest floor (P = 0.005). | | | | |
| --- | --- | --- | --- | --- |
| **Coefficient** | **Estimate** | **Std. Error** | **t** | **p** |
| Urine | 0.226 | 0.082 | 2.757 | 0.005 |
| Intercept (American beech (*Fagus grandifolia*)) | 5.206 | 0.441 | 11.795 | <0.001 |
| Elaeagnus (*Elaeagnus* spp.) | -0.204 | 0.124 | -1.643 | 0.100 |
| Hickory *(Carya spp.)* | 0.579 | 0.194 | 2.971 | 0.003 |
| Ostrya *(Ostrya virginiana)* | -0.610 | 0.112 | -5.427 | <0.001 |
| Chinese privet (*Ligustrum sinense*) | -0.702 | 0.145 | -4.832 | <0.001 |
| Sweetgum (*Liquidambar styraciflua*) | -1.027 | 0.194 | -5.268 | <0.001 |
| Winged elm (*Ulmus alata*) | -0.221 | 0.194 | -1.138 | 0.255 |
|  |  |  |  |  |

| **Supplementary Table 6.** Results from generalized linear model for scrapes created by white-tailed deer (*Odocoileus virginianus*) with urine present (N = 20) in Athens-Clarke County, Georgia, from October 14-November 12, 2024, when exposed to 365 nm ultraviolet (UV) light comparing irradiance of white-tailed deer urine to the surrounding forest floor from 400-554 nm. Urine and adjacent forest floor were exposed to 365 nm UV light and scanned with a PR-650 spectrophotometer to quantify scrape spectral characteristics and investigate photoluminescence. The average irradiance of white-tailed deer urine was greater than the surrounding forest floor (P < 0.001). | | | | |
| --- | --- | --- | --- | --- |
| **Coefficient** | **Estimate** | **Std. Error** | **t** | **p** |
| Urine | 0.595 | 0.034 | 17.083 | <0.001 |
| Intercept (American beech (*Fagus grandifolia*)) | -4.909 | 0.187 | -26.212 | <0.001 |
| Elaeagnus (*Elaeagnus* spp.) | -0.225 | 0.052 | -4.274 | <0.001 |
| Hickory *(Carya spp.)* | 0.316 | 0.082 | 3.829 | <0.001 |
| Ostrya *(Ostrya virginiana)* | -0.387 | 0.047 | -8.103 | <0.001 |
| Chinese privet (*Ligustrum sinense*) | -0.009 | 0.061 | -0.161 | 0.871 |
| Sweetgum (*Liquidambar styraciflua*) | -1.100 | 0.082 | -13.298 | <0.001 |
| Winged elm (*Ulmus alata*) | -0.426 | 0.082 | -5.158 | <0.001 |
|  |  |  |  |  |
